# Severe Parapneumonic Effusion in a Child With Respiratory Syncytial Virus and Streptococcus pyogenes Coinfection

**DOI:** 10.7759/cureus.79080

**Published:** 2025-02-16

**Authors:** Hikaru Sugita, Hiroki Miura, Kazuhiro Horiba, Yoichi Nakajima, Tetsushi Yoshikawa

**Affiliations:** 1 Department of Pediatrics, Fujita Health University School of Medicine, Toyoake, JPN; 2 Laboratory of Bacterial Genomics, Pathogen Genomics Center, National Institute of Infectious Diseases, Tokyo, JPN

**Keywords:** coinfection, next-generation sequencing, parapneumonic effusion, respiratory syncytial virus, streptococcus pyogenes

## Abstract

A four-year-old boy with respiratory syncytial virus (RSV) infection and suspected bacterial coinfection deteriorated despite antibiotic treatment. Intensive care and thoracoscopic debridement were required due to parapneumonic effusion. Despite negative pleural fluid cultures, next-generation sequencing detected group A *streptococcus* (GAS). Even in healthy children without risk factors, RSV infection preceding invasive GAS infection can rapidly deteriorate, making diagnosis difficult.

## Introduction

Since the end of the coronavirus disease 2019 (COVID-19) pandemic, significant outbreaks of pediatric infectious diseases such as respiratory syncytial virus (RSV) and group A *streptococcus* (GAS) infection have been demonstrated around the world [1,2]. RSV is an important pathogen that leads to lower respiratory tract infections in infants and young children and can cause severe complications requiring intensive care unit (ICU) care. Meanwhile, GAS can cause a wide variety of clinical manifestations, from superficial infection to severe invasive GAS infection and post-infectious disease syndromes [2]. With the increasing number of RSV and GAS infections after the relaxation of COVID-19 prevention measures, coinfection with these pathogens might occur, leading to a complex clinical course and severe clinical symptoms.

Here, we describe a pediatric patient with RSV and GAS coinfection who developed a rapidly increasing pleural effusion, necessitating intensive care and thoracoscopic debridement. Detection of GAS sequences from the pleural effusion fluid with next-generation sequencing (NGS) was useful for diagnosing the etiology of parapneumonic effusions in this patient.

This case report is scheduled to be presented as an abstract at the 128th Annual Meeting of the Japan Pediatric Society on April 19, 2025.

## Case presentation

**Figure 1:**
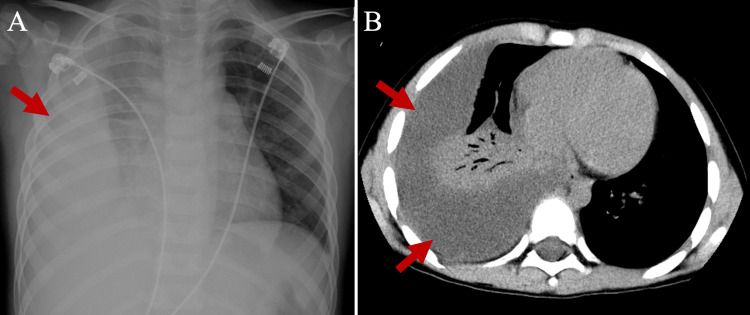
Pre-treatment chest radiography and computed tomography examinations Pre-treatment chest radiograph demonstrated decreased radiopacity in the right lung field, indicative of potential pleural effusion (A). Pre-treatment chest computed tomography revealed significant accumulation of pleural effusion fluid in the right pleural cavity and atelectasis of the right lung (B).

A previously healthy four-year-old boy was admitted to a local hospital with a four-day history of fever, cough, and respiratory distress. He had no prior documented history suggesting immunosuppression. On admission (day 1), white blood cell (WBC) count was elevated at 18,000/μL (reference range: 5,500-15,500/μL), and C-reactive protein (CRP) levels were also elevated at 5.82 mg/dL (reference range: <0.15 mg/dL). The RSV antigen test was positive. Treatment with sulbactam/ampicillin (150 mg/kg/day) was initiated because of the possibility of secondary bacterial infection in the context of RSV infection. Despite treatment, fever persisted, respiratory symptoms rapidly worsened, and chest radiography (Fig. 1A) and computed tomography (CT) (Fig. 1B) on day 4 revealed a substantial right pleural effusion. He was transferred to our hospital.

**Figure 2:**
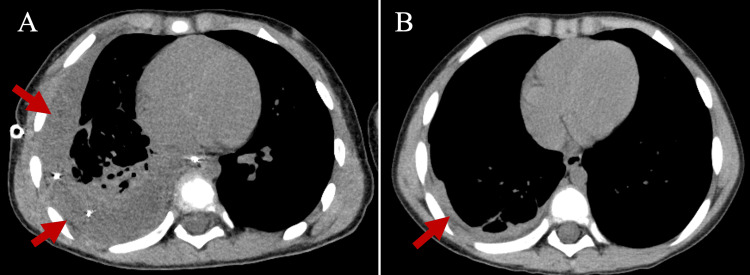
Chest computed tomography findings after treatment interventions Chest computed tomography on hospital day 6, after drainage, revealed persistent pleural effusion in the right pleural cavity and impaired re-expansion of the right lung (A). Chest computed tomography on hospital day 25, after video-assisted thoracoscopic surgery (VATS) for debridement, showed a small amount of residual right pleural effusion, with adequate re-expansion of the right lung (B).

On admission to our hospital (day 4), the patient’s body temperature was 38.7°C and his respiratory rate was 40/min. Nasal oxygen therapy (1 L/min) was required to maintain oxygen saturation of 95%. Breath sounds in the right lung field were markedly decreased. Laboratory tests showed leukocytosis at 19,800/μL (reference range: 5,500-15,500/μL) with a predominance of neutrophils and a markedly elevated CRP level at 12.17 mg/dL (reference range: <0.15 mg/dL). The rapid antigen test for *Streptococcus pyogenes* was positive and the FilmArray Pneumonia Panel (BioFire Diagnostics, Salt Lake City, UT, USA) detected only the RSV genome. Although GAS antigen was detected in this patient, meropenem (120 mg/kg/day) was started at this time because there was no improvement with the initial antibiotic regimen. On day 5, pleural drainage was performed with 180 mL of fluid removed. Pleural fluid analysis revealed a WBC count of 16,910/μL (98.6% neutrophils), glucose <10 mg/dL, total protein of 4.5 g/dL, and lactate dehydrogenase of 4,789 U/L, suggesting a complicated parapneumonic effusion requiring continuous drainage. On day 6, chest CT showed poor drainage and insufficient right lung expansion (Fig. 2A); thus, we decided to perform thoracoscopic debridement. After debridement, the pleural fluid was draining well and the pleural effusion decreased in size. On day 8, the patient’s fever subsided and respiratory condition improved; thus, the endotracheal tube and drainage tube were removed. The patient was transferred to the general ward on day 11. Antibiotic therapy was completed on day 24 and he was discharged from the hospital on day 30. The patient had no respiratory symptoms or abnormal physical examination findings at the time of hospital discharge; however, chest CT on day 25 revealed a small residual right pleural effusion (Fig. 2B).

Immunologic evaluation performed during the convalescent period found normal immunoglobulin concentrations, B- and T-lymphocyte counts, CD4+ and CD8+ cell populations, and neutrophil bactericidal activity.

**Figure 3:**
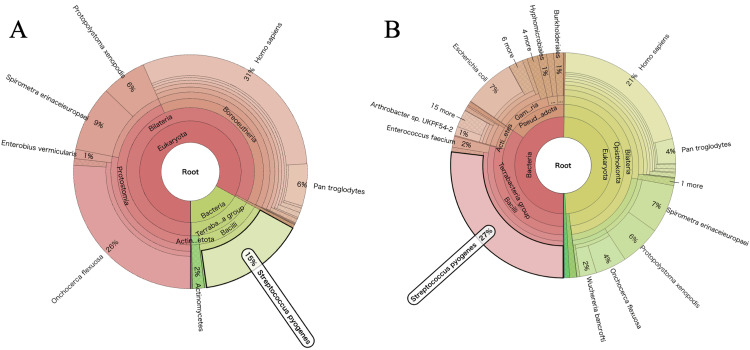
Next-generation sequencing-based genetic analysis of pleural fluid for pathogens DNA analysis of pleural fluid with next-generation sequencing demonstrated a high detection rate for *Streptococcus pyogenes* DNA (A). RNA analysis of pleural fluid with next-generation sequencing demonstrated a high detection rate for *Streptococcus pyogenes* RNA (B).

Two blood cultures performed at the time of admission to each hospital did not isolate any bacteria. Pleural fluid cultures and a FilmArray pneumonia panel of pleural fluid did not reveal any pathogens. Acid-fast bacilli cultures from the sputum were negative. An interferon-gamma release assay for *Mycobacterium tuberculosis *was also negative. Therefore, in order to determine the causative agent of parapneumonic effusion in this patient, pleural fluid was analyzed with NGS. In brief, the DNA sequencing library was prepared with the QIAseq FX DNA Library Kit (Qiagen, Hilden, Germany), and the RNA sequencing library was prepared with the Zymo-Seq RiboFree Total RNA Library Kit (Zymo Research, Irvine, CA, USA). Sequencing was conducted on a NextSeq 2000 analyzer (Illumina, San Diego, CA, USA). The sequencing data was processed with the PATHDET metagenomic pipeline as described previously [3]. Sample quality assessment was performed and human genome-derived data were eliminated. High-homology microorganisms were identified with a BLAST search [4] in the National Center for Biotechnology Information (NCBI; http://www.ncbi.nlm.nih.gov/genomes/). DNA sequencing identified that genes from *Streptococcus pyogenes* constituted 15% of the total DNA (87% from bacterial sources). RNA sequencing showed that comprised 27% of the total RNA (53% from bacterial sources) (Fig. 3A, 3B). In addition, anti-streptolysin O (ASO) titers measured on days 4 and 24 were 72 IU/mL and 386 IU/mL, respectively (reference range: ≤239 IU/mL).

## Discussion

Because this previously healthy four-year-old patient infected with RSV had no risk factors for severe RSV infection such as chronic lung disease or immunodeficiency, physicians did not anticipate the rapid deterioration of the patient’s condition, including a sudden increase in pleural effusion size. Mild leukocytosis and elevated CRP levels were initially observed. Antibiotic treatment was initiated at the time of local hospital admission. Although a rapid GAS antigen test was positive on admission to our hospital, two blood cultures were negative and no bacteria were isolated from the pleural fluid. The second blood culture and pleural fluid samples were collected after antibiotic treatment began; thus, negative bacterial culture results are not completely reliable. In any case, it was impossible to diagnose this patient as having an invasive GAS infection on the basis of microbiological analysis.

Detection of the GAS genome using molecular techniques such as polymerase chain reaction (PCR) has been suggested to be useful for diagnosing invasive GAS infection [5]. It has been demonstrated that the combination of bacterial culture and PCR improved the detection rate of GAS in pleural fluid to 91% [5]. Furthermore, 16S rRNA sequencing with NGS has been demonstrated to be highly sensitive and specific for the identification of pathogens in a variety of clinical samples, including pleural effusion fluid [6,7]. In this case, the GAS genome was detected in the patient’s pleural fluid using a similar NGS analysis, suggesting that the rapid accumulation of pleural effusion fluid in this patient was due to invasive GAS infection. Parapneumonic effusion is a relatively rare complication in healthy children. Its etiology is difficult to determine; however, NGS analysis of pleural fluid might help identify the exact pathogens in parapneumonic effusions in children.

A significant increase in the incidence of invasive GAS infection in children has been demonstrated in the post-COVID-19 pandemic era [8,9]. In the United Kingdom, a substantial increase in the incidence of pediatric pneumonia with pleural effusion or empyema has been demonstrated since 2022 [10], allowing cohort analysis of pediatric GAS pneumonia with parapneumonic pleural effusion in a short period of time [5]. According to the report, the majority of the 146 patients were between one and four years old, the same age as the current patient. Furthermore, contrary to expectations, previously healthy children accounted for 88% of all patients. RSV was the second most common virus in that study. Non-pharmaceutical interventions implemented during the COVID-19 pandemic suppressed various pediatric infections, including GAS and RSV [1,2], which might be associated with recent epidemiological changes in pediatric GAS pneumonia with parapneumonic pleural effusion. This case represents a valuable example suggesting that, even in relatively older children who are otherwise healthy and at low risk for severe disease, RSV infection can occasionally lead to severe invasive infection when there is a secondary GAS infection, posing diagnostic challenges. As detailed information on the entire clinical course of such patients is limited, this case report provides important information for clinicians.

## Conclusions

We describe a previously healthy four-year-old boy with RSV infection who presented with severe GAS pneumonia and parapneumonic pleural effusion requiring intensive care. Although microbiological examinations such as bacterial cultures of blood and pleural effusion fluid were unable to identify the etiology of this severe complication, NGS of pleural fluid was useful for identifying the etiological agent. Unusual outbreaks of RSV and GAS infections that occurred after the COVID-19 pandemic might have increased the risk of severe complications due to co-infection with both pathogens, warranting close attention.

## References

[1] Chuang YC, Lin KP, Wang LA, Yeh TK, Liu PY (2023). The impact of the COVID-19 pandemic on respiratory syncytial virus infection: a narrative review. Infect Drug Resist.

[2] (2024). Increased incidence of scarlet fever and invasive Group A Streptococcus infection - multi-country. https://www.who.int/emergencies/disease-outbreak-news/item/2022-DON429.

[3] Horiba K, Torii Y, Aizawa Y (2022). Performance of nanopore and illumina metagenomic sequencing for pathogen detection and transcriptome analysis in infantile central nervous system infections. Open Forum Infect Dis.

[4] Morgulis A, Coulouris G, Raytselis Y, Madden TL, Agarwala R, Schäffer AA (2008). Database indexing for production MegaBLAST searches. Bioinformatics.

[5] Lees EA, Williams TC, Marlow R (2024). Epidemiology and management of pediatric Group a streptococcal pneumonia with parapneumonic effusion: an observational study. Pediatr Infect Dis J.

[6] Church DL, Cerutti L, Gürtler A, Griener T, Zelazny A, Emler S (2020). Performance and application of 16S rRNA gene cycle sequencing for routine identification of bacteria in the clinical microbiology laboratory. Clin Microbiol Rev.

[7] Moorlag SJ, Coolen JP, van den Bosch B, Jin EH, Buil JB, Wertheim HF, Melchers WJ (2023). Targeting the 16S rRNA gene by reverse complement PCR next-generation sequencing: specific and sensitive detection and identification of microbes directly in clinical samples. Microbiol Spectr.

[8] Abo YN, Oliver J, McMinn A (2023). Increase in invasive group A streptococcal disease among Australian children coinciding with northern hemisphere surges. Lancet Reg Health West Pac.

[9] van Kempen EB, Bruijning-Verhagen PC, Borensztajn D (2023). Increase in invasive Group a streptococcal infections in children in the Netherlands, a survey among 7 hospitals in 2022. Pediatr Infect Dis J.

[10] Guy R, Henderson KL, Coelho J (2023). Increase in invasive group A streptococcal infection notifications, England, 2022. Euro Surveill.

